# Transcriptomics analysis of host liver and meta-transcriptome analysis of rumen epimural microbial community in young calves treated with artificial dosing of rumen content from adult donor cow

**DOI:** 10.1038/s41598-018-37033-4

**Published:** 2019-01-28

**Authors:** Wenli Li, Andrea Edwards, Christina Riehle, Madison S. Cox, Sarah Raabis, Joseph H. Skarlupka, Andrew J. Steinberger, Jason Walling, Derek Bickhart, Garret Suen

**Affiliations:** 1 The Cell Wall Utilization and Biology Laboratory, US Dairy Forage Research Center, USDA ARS, Madison, WI 53706 USA; 2 Department of Biology, University of Wisconsin-Madison, Madison, WI 53706 USA; 3 Department of Genetics, University of Wisconsin-Madison, Madison, WI 53706 USA; 4 Department of Bacteriology, University of Wisconsin-Madison, Madison, WI 53706 USA; 5 Department of Medical Sciences, School of Veterinary Medicine, University of Wisconsin-Madison, Madison, WI 53706 USA; 6 Cereal Crops Research Unit – USDA, 502 Walnut Street Madison, Madison, WI 53726 USA

**Keywords:** Gene expression analysis, Functional genomics

## Abstract

In mammals, microbial colonization of the digestive tract (GIT) occurs right after birth by several bacterial phyla. Numerous human and mouse studies have reported the importance of early gut microbial inhabitants on host health. However, few attempts have been undertaken to directly interrogate the role of early gut/rumen microbial colonization on GIT development or host health in neonatal ruminants through artificial manipulation of the rumen microbiome. Thus, the molecular changes associated with bacterial colonization are largely unknown in cattle. In this study, we dosed young calves with exogenous rumen fluid obtained from an adult donor cow, starting at birth, and repeated every other week until six weeks of age. Eight Holstein bull calves were included in this study and were separated into two groups of four: the first group was treated with rumen content freshly extracted from an adult cow, and the second group was treated with sterilized rumen content. Using whole-transcriptome RNA-sequencing, we investigated the transcriptional changes in the host liver, which is a major metabolic organ and vital to the calf’s growth performance. Additionally, the comparison of rumen epimural microbial communities between the treatment groups was performed using the rRNA reads generated by sequencing. Liver transcriptome changes were enriched with genes involved in cell signaling and protein phosphorylation. Specifically, up-regulation of SGPL1 suggests a potential increase in the metabolism of sphingolipids, an essential molecular signal for bacterial survival in digestive tracts. Notably, eight genera, belonging to four phyla, had significant increases in abundance in treated calves. Our study provides insight into host liver transcriptome changes associated with early colonization of the microbial communities in neonatal calves. Such knowledge provides a foundation for future probiotics-based research in microbial organism mediated rumen development and nutrition in ruminants.

## Introduction

Young calves are born with an under-developed reticulo-rumen^1^ and are forced to behave like monogastrics while fed milk-based diets, which are digested in the abomasum. Later in life, the calves transition to dry and forage based diets, which requires further development of the rumen from the esophageal groove and the colonization of an associated microbial community^2,3^. During this transition, the optimal early development of the rumen is critical to ensure animal health, productivity, and related economic benefits to the producers. The development of the rumen during this transition involves three simultaneous components. (1) Firstly, there must be an anatomical development of the rumen, including growth in rumen volume and rumen papillation^4^. There are three phases involved in the anatomical development of rumen, including non-rumination (0–3 weeks); transitional phase (3–8 weeks) and rumination (from 8 weeks on)^5^. (2) Simultaneously, there is a colonization and establishment of the microbial community in the developing rumen^6,7^. (3) Finally, there is a functional development of fermentation capacity and enzyme activity in the rumen lumen and epimural layers^8^. These developments lead to a major change in nutrients delivered to the intestines and liver, and subsequent peripheral tissue of the animal^9^.

Gut microbial colonization is an important process that accompanies the rumen development process^10^. In ruminant livestock, rumen microbes provide 70% of the daily energy requirement of ruminants^11^. Studies have shown that different feeding management regimes may be associated with different microbial populations establishing in the rumen of young ruminants^7,12–14^. In the rumen of young lambs fed with forage, a different composition of microbial community was observed in comparison to the ones fed with concentrate, and this difference in microbial composition persisted over months. Artificially reared young ruminants have different protozoa and microbial populations as compared to those reared with their mothers, raising the possibility of a beneficial effect by direct microbial inoculation by the dam^12,15^. Moreover, despite a reported host-specificity in the rumen microbiome community, pre-weaning diet and feeding methods have been reported to have pronounced and long-lasting impacts on rumen microbial composition^12,13^. In comparison to calves weaned conventionally (at 6 weeks), calves introduced with solid feed early (at 3 weeks) showed much greater microbial abundance in the rumen^16^.

In addition to the effect on microbial communities observed using nutritional manipulation, direct feeding of live microorganisms to ruminants has also been reported to be advantageous^17,18^. Inoculation with fresh rumen fluid into rumens in early weaned lambs improved average daily gain and digestibility^19^. By adding a fungal probiotic, Theodorou *et al*.^20^ observed increased intake and live weight gain in calves at weaning. In adult ruminants, such artificial manipulation is short-lived and there is strong evidence of host specificity after mature rumen microbial colonization^21^. Early dietary experience contributes to a more pronounced and lasting effect on the rumen microbial composition^22^ and as a result various processes may be involved during early life adaptation, including neuroendocrine, morphological and physiological changes^23^. The most critical is the maturation of the immune system in the host modulated by early colonization events of microbes^24^. Collectively, these studies led to a hypothesis of microbial programming in early life.

Microbial colonization has an effect on the host’s innate immune response^25,26^. Tracheal antimicrobial peptide (TAP) gene, a *β*-Defensin gene^27^, is a front-line protectant against pathogens, and TAP expression has been associated with inflammation^28^. Elevated expression of TAP in tracheal epithelial cells has been observed after introduction of bacteria in cattle^29^. Additional roles of TAP include providing a link between innate and adaptive immune responses against microbial invasions^30^. TAP is believed to play a critical role in microbicidal activity against bacterial pathogens causing bovine respiratory disease. Notably, the *β*-defensin gene family in cattle was reported to have the most diverse repertoire so far identified^31^. In sheep, peak expression of *β*-defensin 1*(also referred to as oBD1*) from tongue to colon, and *β*-defensin 2 (*oBD2)* in the distal ileum were identified in the first 6–8 weeks of life^32^, with substantial expression in the digestive tract in pre-natal lambs^33^. Toll-like receptors (TLRs) are another class of host proteins with important roles in recognizing commensal and pathogen associated molecular patterns (PAMPs) from the gut flora. The utility of TLR signaling has been demonstrated in the maintenance of tight junctions between epithelial cells, and antimicrobial peptide expression^34^. These studies suggest that the genes involved in host innate immunity are critical players in managing a complex interface between host immune surveillance and the newly colonized rumen microbial community.

Several studies showed that initial microbial colonization occurs immediately after birth, with substantial colonization by critical bacterial phyla and genera in the first few days of life^7,12,35^. One of these studies found that, at birth, Methanogens abd fibrolytic bacteri were already present in the rumen^35^. At day two, the rumen microbial community was mainly composed of Proteobacteria, Bacteroidetes and Prevotella^7^. And an abrupt change was observed in the ruminal bacterial between days 2 and 3 and until day 12, with dominant genera being *Bacteroides*, *Prevotella*, *Fusobacterium* and *Streptococcus*^7^. Numerous human and mouse studies have reported the importance of early gut microbiota on host health, energy intake and storage^36^. However, few attempts have been undertaken to understand the role of early gut/rumen colonization on GIT development or host health in neonatal ruminants^37^. Thus, molecular mechanistic changes associated with bacterial colonization are largely unknown. In this study, we aim to characterize the host physiological changes resulting from the dosing of exogenous rumen content to post-natal calves. Specifically, we used a tissue transcriptome approach to study the host transcriptome changes in the liver that are vital to nutrient absorption. Additionally, we studied the meta-transcriptomics of the rumen microbial community using rRNA reads generated by RNA-sequencing.

## Results

### Bacterial community diversity of administered rumen inoculum

**Figure 1:**
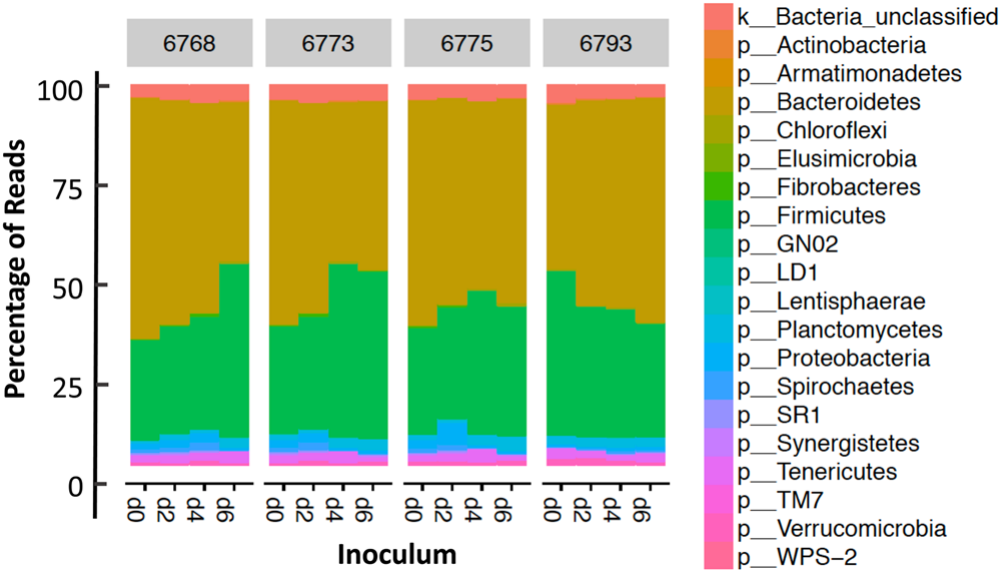
Microbial community composition analysis using targeted 16S rRNA genomic sequencing. Sequencing reads were generated by next-generation sequencing of targeted, genomic amplicon 16S rRNA gene v4 region.

A total of 16 administered inoculum samples (collected at four times points immediately prior to the dosing of each treated calf) were subjected to bacterial composition analysis using next-generation sequencing. After sequence cleanup in mothur^38^, a total of 669,108 high quality reads were obtained for these samples (average per sample 51,469 ± 4,823 SEM). Good’s coverage was greater than 97% for all samples. Following normalization to 10,000 sequences per sample, observed bacterial community composition in all the rumen inoculum was comparable to each other. In Fig. 1, inoculum samples were grouped by the calf ID to which they were administered to. Within each calf ID, administered inoculum samples were labeled by the time they were collected (0, 2, 4 and 6 weeks from birth). In all samples, *Bacteroidetes* was the most abundant phylum (53.31 ± 1.85% SEM) followed by Firmicutes (34.32 ± 1.82% SEM) (Fig. 1**)**.

### RNA quality, sequencing reads and total number of expressed genes

For liver samples, the average RIN (RNA integrity number) for extracted RNAs was 8.43 ± 0.09 (s.e.). The average RIN for RNAs extracted from rumen papillae was 9.08 ± 0.16 (s.e.). An average of five million rRNA reads, 5,072,494 ± 983,485 (s.e.), were obtained for microbial classification analysis for each rumen epimural microbial community. Total number of RNA sequencing raw reads for liver samples ranged from 69 M to 81 M, with an average of 76.8 M ± 1.29 M (s.e.). Total number of expressed genes ranges from 13,669 to 14,287 (fpkm cutoff > = 5) (Supplemental Table 1).

### Differentially expressed genes between treated and untreated calves

In liver tissue, a total of 338 genes showed significant differential expression (fold change > = 1.5, and adjusted *p*-value < = 0.05). In the treated group (in comparison to the control group) 194 were up-regulated and 144 of them were down-regulated. (Supplemental Table 2). Gene ontology (GO) term enrichment analysis was performed using DAVID^39^, with *Bos taurus* as the reference. Top GO terms enriched by differentially expressed genes (DEGs) identified in liver transcriptomes include the following (Table 1): cellular component organization and biogenesis (GO:0071840, *p*-value = 0.02), single-organism metabolic process (GO:0044710, *p*-value = 0.01), regulation of signaling (GO:0023051, *p*-value = 0.04), positive regulation of nitrogen compound metabolic process (GO:0051173, *p*-value = 0.04), peptidyl-tyrosine phosphorylation (GO:0018108, *p*-value = 0.01), and innate immune response (GO:0045087, *p*-value = 0.05).

**Table 1:** GO terms for DEGs in liver.

Category	Term	Count	%	*p*-value
GOTERM_BP_ALL	GO:0008152~metabolic process	115	43.89	0.049
GOTERM_BP_ALL	GO:0071840~cellular component organization or biogenesis	73	27.86	0.026
GOTERM_BP_ALL	GO:0048518~positive regulation of biological process	61	23.28	0.026
GOTERM_BP_ALL	GO:0044710~single-organism metabolic process	49	18.70	0.012
GOTERM_BP_ALL	GO:0048583~regulation of response to stimulus	44	16.79	0.022
GOTERM_BP_ALL	GO:0044765~single-organism transport	38	14.50	0.037
GOTERM_BP_ALL	GO:0023051~regulation of signaling	37	14.12	0.042
GOTERM_BP_ALL	GO:0022607~cellular component assembly	34	12.98	0.024
GOTERM_BP_ALL	GO:0043933~macromolecular complex subunit organization	33	12.60	0.008
GOTERM_BP_ALL	GO:0012501~programmed cell death	30	11.45	0.001
GOTERM_BP_ALL	GO:0010605~negative regulation of macromolecule metabolic process	29	11.07	0.024
GOTERM_BP_ALL	GO:0051173~positive regulation of nitrogen compound metabolic process	23	8.78	0.043
GOTERM_BP_ALL	GO:0051130~positive regulation of cellular component organization	22	8.40	0.003
GOTERM_BP_ALL	GO:0048878~chemical homeostasis	19	7.25	0.004
GOTERM_BP_ALL	GO:1902533~positive regulation of intracellular signal transduction	17	6.49	0.009
GOTERM_BP_ALL	GO:0097190~apoptotic signaling pathway	15	5.73	0.002
GOTERM_BP_ALL	GO:0055082~cellular chemical homeostasis	13	4.96	0.014
GOTERM_BP_ALL	GO:0018108~peptidyl-tyrosine phosphorylation	8	3.05	0.010
GOTERM_BP_ALL	GO:0055074~calcium ion homeostasis	8	3.05	0.043
GOTERM_BP_ALL	GO:0045785~positive regulation of cell adhesion	8	3.05	0.048
GOTERM_BP_ALL	GO:0046883~regulation of hormone secretion	7	2.67	0.008
GOTERM_BP_ALL	GO:0070371~ERK1 and ERK2 cascade	7	2.67	0.023
GOTERM_BP_ALL	GO:0097191~extrinsic apoptotic signaling pathway	7	2.67	0.028
GOTERM_BP_DIRECT	GO:0045087~innate immune response	7	2.67	0.050

**Figure 2:**
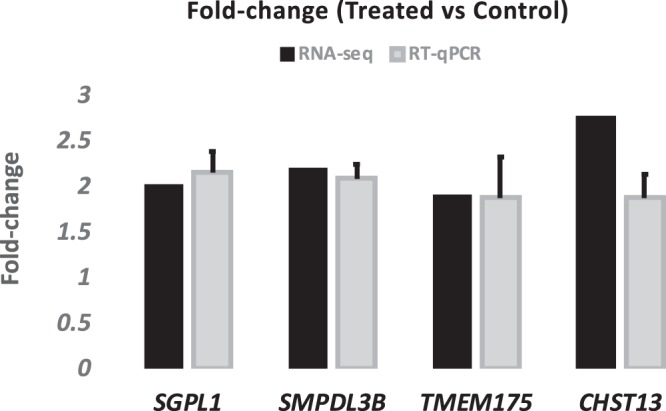
Fold-change (Treated vs. control) of four genes determined by both RT-qPCR and RNA-seqencing methods.

Annotation of the top 25 most upregulated genes include those associated with immune response and sphingolipid metabolism. Eight genes were involved in the innate immune response (Supplemental Table 3). The expression profile of homologs of these genes were investigated using tissue-specific gene expression data in cattle (*Bos taurus*) from the Gene Expression Atlas databases^40^ (https://www.ebi.ac.uk/gxa/home). And due to the comprehensive list of tissues included in the database, human tissue expression database (GTEx project, (https://www.gtexportal.org/home/) was also checked. Among these, *SMPDL3B* showed predominant expression in human GI tissue types (Supplemental Fig. 1a) and high expression in cattle liver (Supplemental Fig. 1b). Fibrinogen beta chain (*FGB*) and fibrinogen gamma chain (*FGG*) showed exclusive expression in human liver (Supplemental Fig. 2a) and high expression in cattle liver (Supplemental Fig. 2b). The rest of the genes showed nearly universal expression across investigated tissues. For RT-qPCR results, all of four of the chosen genes were confirmed by our RT-qPCR results, with significant up-regulation in treated animals (Fig. 2).

### Results of taxonomic classification of rumen wall microbial community

**Figure 3:**
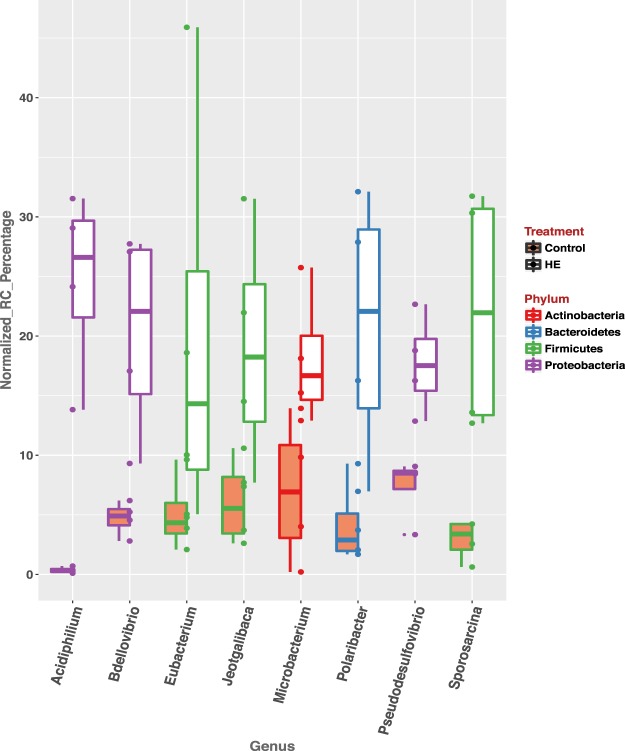
The abundance of eight genera are significantly higher in treated group (*p*-value < 0.05), in comparison to control group. rRNA sequencing reads mapped to each genera by Kraken were used to calculate the normalized read counts.

**Figure 4:**
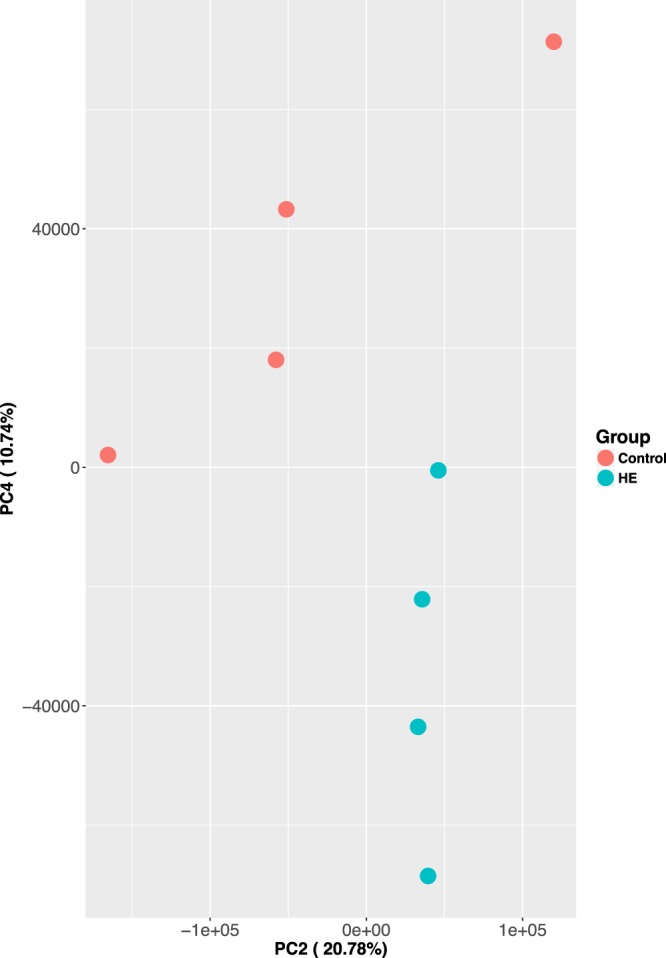
PCA plot using normalized, genus level read counts. Control and treated animals separate along PC2 and PC4, accounting 31.52% of the overall differences. Rumen microbial rRNA reads were obtained by rumen papillae tissue RNA-seq. Genus classification was done using Kraken.

The average classification rate is 97.9 ± 1.4 (s.e.). Among the top 10% most abundant taxa at genus level, 29 of them are shared between the calves from control and treated groups; four of them are unique to the control group (*Aeromonas*, *Alistipes*, *Desulfotomaculum* and *Candidatus Arthromitus*), and three of them are unique to the treated group (*Corynebacterium*, *Thermoanaerobacterium* and *Algoriphagus*). The abundance of 8 genera are significantly higher in the treated group (*p*-value < 0.05). These are: *Acidiphilium*, *Jeotgalibaca*, *Polaribacter*, *Pseudodesulfovibrio*, *Bdellovibrio*, *Microbacterium*, *Eubacterium*, *Sporosarcina*. And they belong to four phyla: *Firmicutes*, *Proteobacteria*, *Bacteroidetes* and *Actinobacteria* (Fig. 3). PCA analysis revealed that the calves treated with rumen-inoculum from high-efficiency donor (HE) separated from control animals along PC2 and PC4 **(**Fig. 4**)**, counting for 31.52% of the overall differences between control and treated groups.

## Discussion

### Genes with elevated expression changes and GO term annotation in liver

GO term annotation for the genes with most significant fold change between treated and control groups mainly fell into two groups: host-level molecular change in response to infection, including phosphorylation and cell signaling transduction; and mechanisms that facilitate bacterial establishment. Host protein phosphorylation during bacterial infection has been reported previously^41,42^. As the most widespread mechanism of post-translational modification, protein phosphorylation is reversible, and is commonly used to activate or deactivate cellular processes by switching enzyme activities “on” or “off”. During microbial colonization in treated calves, it is likely that phosphorylation-based cellular signaling sent from the host, contributes to immunity and to restricting foreign microbial invasion. Future protein phosphorylation based studies will most likely help identify the key protein phosphorylation events that co-occur with microbial invasion and establishment.

Among the major groups of microbial inhabitants in the rumen, bacteria are the most responsible for fermenting feed into an absorbable energy source by cattle^21^. In dairy cattle, *Bacteroidetes* is a dominant bacteria phylum in the rumen^43,44^, with a predominant presence in pre-weaned calves^45^. Several beneficial roles of *Bacteroides* have been reported and they include: contributing to the normal development of the gastrointestinal tract (GIT)^46^, interacting with the immune system to induce T-cell mediated responses^47,48^, and limiting the GIT colonization by potential pathogenic bacteria^49^.

Notably, we observed significant up-regulation of SGPL1 in liver transcripts. SGPL1 encodes Sphingosine 1-phosphate (S1P), which is a polar sphingolipid metabolite that regulates cell migration, differentiation, survival and complex physiological processes^50^. Sphingolipids entail diverse signal transduction, and are involved in stress response pathways with profound physiological impacts as demonstrated in a variety of eukaryotic cell types^51^. Quite interestingly, sphingolipid is reported to be essential for *Bacteroides* physiology, enabling them to perform functions related to symbiosis in the intestines^52^, and to play an important role in the development of immunological tolerance to commensal microbiota^49^. Given its diverse function in dealing with stress, sphingolipid-mediated signaling is an ideal mechanism for both the host and foreign bacteria to adapt and survive. Thus, we hypothesize that artificial dosing of rumen contents introduced a rich influx of Bacteroidetes into the GIT of newborn calves. These newly introduced bacteria imposed a threat to the host while, in the meantime, experienced intensive stress from the GIT of the host. As a metabolic organ, the metabolic activity of liver is controlled by many signals produced by the host. When the host produces excess amount of sphingolipid as an adaptive response to stress, increased metabolism of sphingolipid is observed in the liver.

### Early dosing and host immune response

At the early stage of microbial invasion, the host immune response provides the frontline protection against newly introduced microbial species. Gut microbiota has a critical role in the development of both innate and adaptive components of the mucosal immune system^10^. Our RNA sequencing work provided evidence that immunity related genes responded to the alterations in gut microbiota with elevated expression levels in the liver. This finding suggests that the calves are coping to the exogenous bacterial introduction by turning on their immune and defense responses. Accordingly, we observed seven genes with substantial expression changes that are each involved in the adaptive and innate immune responses. Notably, *SMPDL3B*, encoding a membrane-modulating enzyme showed 2.5 fold change in expression in our experiment. Heinz *et al*.^53^ indicated that this gene may function at the interface of membrane biology and innate immunity. Interestingly, the human homolog of this gene shows predominant expression in an array of GI tissues, indicating a potentially significant role of this gene in the molecular mechanisms of GI physiology. Two genes, FGG and FGB, showed exclusive expression in liver, reflected by the human tissue-specific gene expression data. Aside from their roles in the adaptive immune response, these two genes were also shown to be involved in inflammatory pathways, with greatly increased expression in hepatocytes during inflammatory stress^54^.

Our study suggests that genes involved in the immune response, anti-inflammatory response and signaling are poised to provide both innate and adaptive immune responses and ‘tolerance’ to the first colonizers of the rumen. Immune responses consume a great deal of energy from the host with the desired outcome of destroying foreign intruders. Further studies may focus on targeted regulation of immunity- and anti-inflammation-associated genes during the neo-natal stage following artificially dosing rumen contents. For example, comparing the colonization rates of an exogenously introduced bacterial community in controls that do not demonstrate host gene regulation to those individuals comprising a treated group that does exhibit gene regulation. Such studies will undoubtedly provide more insights into the molecular mechanisms responsible for early microbial establishment in cattle.

In our experiment, we observed that innate immune response related genes showed significant up-regulation in calves dosed with the rumen inocula obtained from the high-milk production cow compared to the control calves. And yet, we did not observe clinical signs associated with pathological infection, *e.g*., no obvious increase in body temperature in treated calves in comparison to the control calves. Additionally, calves dosed with adult cow rumen content did not appear to have clinical signs consistent with enteric infections. Quite interestingly, studies that administered probiotics to calves have reported several beneficial effects, *e.g*., decreased incidence of diarrhea, and overall weight gain and increased efficiency in feed conversion^18^, which likely occurs due to the effect of promoting colonization of beneficial bacteria, while decreasing the occurrence of detrimental bacteria^55^. Thus, there might be a beneficial effect on calf health through early artificial dosing using adult cattle rumen content, which warrants further in-depth investigation.

### Successful graft of exogenous inoculation of rumen content

Rumen content used in our dosing experiment was from an adult cow, within which the rumen microbial population has adapted to the host GIT environment and reached a homeostasis. When introduced to the newborn calf, this microbial population likely faced a new GIT environment, which undoubtedly elicited immune responses from the host. The first question regarding artificial dosing is whether these newly introduced rumen microbiota can survive in the new environment. Using the microbial rRNA reads extracted from our RNA-sequencing experiments, we identified that eight genera, belonging to four phyla, had significant increases in abundance in treated calves in comparison to control calves. Notably, three of these phyla, Bacteroidetes, Firmicutes and Proteobacteria have been reported as the most abundant microbial phyla in the rumens of adult dairy cattle^56^, suggesting that this inoculation may be hastening the development of a mature rumen microbial community in treated calves. Since the inoculum samples used for the treatment group were extracted from an adult cow, our meta-transcriptomic analysis provided strong evidence of successful graft of these adult cow-originated microbial phyla. Additionally, we observed significant up-regulation of genes involved in sphingolipid metabolism. These data indicate that the newly introduced microbiota have colonized the nascent rumen of the calf and actively interacted with the host GIT epithelial cells. Given existing evidence suggesting the host-specificity of cattle rumen microbial content^21^, follow-up studies may focus on determining the exact microbial species that successfully colonize within the calves. Gained knowledge might provide guidance into better management of feed during artificial dosing, *e.g*., variety of nutrients that facilitate establishment of beneficial microbial species, and selective dosing of a combination of microbial species to increase retention time and final colonization.

### Future perspectives

Our study represents a snap-shot of the host liver transcriptome changes in response to artificial dosing of rumen microbiota. Results from this study indicated that significant host liver transcriptome changes are triggered by the newly introduced microbial community. For future follow-up studies, comparative analyses at different time points during the artificial dosing experiments will provide more quantitative measures regarding how and when certain transcripts in the host are affected by exogenous, artificially dosed microbes. Knowledge obtained through these studies will most likely facilitate consistent and successful probiotic treatment of exogenous microbial species that are beneficial to the host. Additionally, further investigation into artificial dosing induced host immune responses may be leveraged as a means to promote health and productivity of newborn calves.

## Methods

### Experimental Design

This experiment was approved by University of Wisconsin-Madison, Institutional Animal Care and Use committee (IACUC). All animals involved in this study were fed and watered according to the herd standard practices used at the USDA Dairy Forage Research Center farm throughout the experiment. Animal care and use, and all methods involved in this study were performed in accordance with the relevant guidelines and regualtions by the Animal Wellfare Act from US Department of Agriculture and by the Federation of Animal Science Societies.

This study covered only a subset of a larger experiment. For the larger experimental design, two adult cows, previously identified as high milk production efficiency (HE) cow, and low milk production efficiency (LE) cow, as described in Jewell *et al*.^56^, were used as donors. The main objective of this study was to discern the impact of artificially dosed, live-microbes on the rumen epimural microbial community and liver transcriptome. For this purpose, one HE cow was used as the donor in this study. The diet composition of this donor cow was described in detail in Jewell *et al*.^56^. For the treated groups, the calves were dosed with the inocula obtained from this one donor cow’s rumen content (HE group). For the control group, they were dosed with sterilized inocula (control group).

### Rumen inocula preparation and dosing

The inocula obtained from the donor cow were prepared as follows: rumen liquids and lightly squeezed solids were collected from the medio-ventral region of the rumen in a 3:1 ratio by volume. The mixture was immediately blended under CO_2_ for 1 minute, then squeezed through four layers of cheesecloth to remove large particles. Inoculum samples were taken right after blending and placed in a 15 mL conical tube (Corning, Oneonta, NY). For treatment cohorts, fresh inoculum was used same-day. The control inocula were prepared by autoclaving the combined inocula (50% from each) from the HE donor cow and the LE donor cow. For both treatments, doses were administered to bull calves within 3 days after birth, then 2, 4, and 6 weeks following the initial dosing. 50 mL of inoculum was delivered by oral gavage, then followed with 50 mL of sterile McDougall’s buffer to ensure complete clearance of the inoculum from the dosing tube.

### DNA extraction and 16S Metagenomics analysis of HE rumen inoculum

Immediately prior to dosing, a portion of freshly prepared HE inoculum samples was collected and stored in −80 °C, at days 0, 2, 4 and 6. A total of 16 inoculum samples were collected, as four biological replicates were retrieved at each time point. To prepare for DNA extraction, frozen inoculum samples were thawed in a room temperature water bath. DNA was extracted using an adaptation of the methods described previously^56,57^. In short, cells were pelleted at 5000 g for one hour, then resuspended in approximately 2 mL of chilled DNA extraction buffer. 1 mL of this resuspension was transferred to a 2 mL screw-cap tube with 0.5 g for 0.1 mm zirconium beads, 50 uL 20% SDS, and 700 uL cold equilibrated phenol. This mixture was subjected to bead beating for 2 minutes on a tabletop bead beater (Mini Bead Beater, Biospec Products, Bartlesville, OK), then heated in a 60 °C water bath for 10 minutes before another 2 minutes of bead beating. The mixture was then centrifuged for 10 minutes at 4 °C on a tabletop centrifuge at max speed (~15,000 rpm, Microfuge 20 R, Beckman Coulter, Brea, CA). The aqueous layer was washed 2–4 times with cold equilibrated phenol until the white lipid layer disappeared before precipitation of DNA in a mixture of 0.1 vol 2 M Na acetate and 0.6 vol isopropanol overnight. DNA was pelleted in a tabletop centrifuge for 20 minutes at 4 °C with max speed. The pellet was washed with 70% ethanol, then dried overnight. Pellet was resuspended in 100 μL of elution buffer (Invitrogen, Thermo Fisher Scientific, Waltham, MA).

To amplify the 16S rRNA gene, universal primers flanking the variable 4 (V4) region were used^58^. For one reaction per sample, 25 ng of template DNA, 5 pmol of each of the forward and reverse primers, and 12.5 μL of 2X HotStart ReadyMix (KAPA Biosystems, Wilmington, MA), and water to a total volume of 25 μL were used. Cycling conditions were as follows: initial denaturation of 95 °C for 3 minutes, 25 cycles of 95 °C for 30 seconds, 55 °C for 30 seconds, and 72 °C for 30 seconds, and a final extension at 72 °C for 5 minutes. Size exclusion was performed by gel electrophoresis on a 1.0% low-melt agarose gel (National Diagnositcs, Atlanta, GA), followed by gel extraction of amplified DNA using a ZR-96 Zymoclean Gel DNA Recovery Kit (Zymo Research, Irvine, CA). Extracted DNA was quantified via a 96-well protocol using manufacturer’s instructions with the Quant-iT™ dsDNA High-Sensitivity Assay Kit, using reagents from a Qubit® dsDNA Assay Kit (Thermo Fisher Scientific, Waltham, MA). Reactions were read on a Synergy 2 Multi-Mode Reader (BioTek, Winooski, VT) following a programmed 3 second shaking period and a 2 minute incubation at 22 °C. Amplified DNA was equimolar pooled, combined with 10% PhiX control DNA, and sequenced using the MiSeq. 2 × 250 v2 kit (Illumina, San Diego, CA) with custom sequencing primers as described by Kozich *et al*.^58^.

The program mothur v.1.38.1 was used for further sequence processing^38^, following a protocol developed from Kozich *et al*.^58^, as described in Weimer *et al*.^59^. In short, paired-end sequences were assembled into continuous segments and poor-quality sequences were removed. The SILVA 16S rRNA gene reference alignment database v128^60^ was used to screen for alignment to the v4 region. Preclustering was performed (diffs = 2) to reduce error and computational load, and chimeric sequences were removed (UCHIME^61^). The 2013 release of the GreenGenes database^62^ was used to classify sequences with a bootstrap value cutoff of 80. Sequences classifying to cyanobacteria, mitochondria, Eukarya, or Archaea were removed. Bacterial sequences were grouped into operational taxonomic units (OTUs) with 97% sequence similarity. Good’s coverage^63^ was calculated in mothur. OTU counts were normalized to 10,000 sequences per sample, and normalized OTU counts were used for further analysis. Stacked bar plots were generated in R version 3.4.3 using the package phyloseq v1.22.3^64^.

### Calf tissue collection

Liver and rumen papillae were included in this study. These tissues were collected immediately after animal sacrifice. Upon collection, tissues were rinsed in PBS to remove feed particles if present, and cut with sterilized scalpels into 4–5 mm^2^ fragments and put into Eppendorf safe-lock tubes (Eppendorf North America, Hauppauge, NY). Collected tissues were flash frozen in liquid nitrogen and stored at −80 °C for long-term storage.

### RNA extraction, quantification and whole transcriptome sequencing

Tissues were homogenized into fine powders in liquid nitrogen using a mortar and pestle. RNAs were extracted using miRNeasy kit (Qiagen, US) following manufacturer’s protocol. Quality of extracted RNAs were assessed using Bioanalyzer RNA 6000 nano kit (Agilent Technologies, US). RNA samples with RIN value > = 8 were pursued for RNA quantification using Qbit (Thermo Fisher, US). RNA-sequencing library preparation was done using Illumina TruSseq ribo-zero gold kit following manufacturer’s instructions. For each sample, 1 μ*g* of total RNA was used for sequencing library preparation. After libraries were prepared for each sample, quantification of library was performed using Kapa quantification kit (Kapa systems) using ABI7300 instrument. Libraries were further normalized to ensure equal quantity before sequencing. 2 × 150 bp paired-end reads were obtained using Illumina NextSeq 500 instrument with high-output kit.

### Mapping of RNA sequencing raw reads and differential gene expression analysis

Quality of raw reads were checked using FastQC (https://www.bioinformatics.babraham.ac.uk/projects/fastqc/). For sequence alignment, NCBI UMD3.1, *Bos taurus* reference genome was used. Raw reads from all whole transcriptome RNA-seq libraries were aligned using a two-step alignment approach. First, Tophat2^65^ was used with the following settings: ‘-r 70–mate-std-dec 90′ for paired-end reads from Illumina RNA-seq. Second, unmapped reads from step one were realigned with Bowtie2^66^ using the “–very-sensitive-local” method. The genome annotation file (NCBI, UMD3.1) downloaded from Tophat website (http://ccb.jhu.edu/software/tophat/igenomes.shtml) was used as reference. Genes shorter than 150 bp were excluded from the GTF file. Raw reads shorter than 50 bps were excluded from the alignment process. Raw read counts for each gene were obtained using HTSeq (v0.6) HTseq.^67^. Combined (Tophat + bowtie2) sequence alignment generated by the two-step alignment approach served as input file for HTSeq. The expression level of mRNAs in each sample were normalized to fpkm using cufflinks^68^. Normalized fpkm values were used to assess gene expression profiles for each sample. Total number of expressed genes were calculated using fpkm cutoff value of 5.

DEG analysis was performed using the R/Bioconductor package DESeq 2^69^ with raw read counts from RNA-Seq. Genes with less than ten normalized read counts were excluded from further analysis. Read count normalization was performed using the regularized logarithm (rlog) method provided in DESeq2. DEGs were determined by adjusted *p-*value (cutoff of 0.05) and the fold change (cutoff of 1.5) by DESeq2. Gene function annotation and pathway analysis were performed using DAVID^39^ and stringDB^70,71^.

### Taxonomic classification of rumen wall microbial community using rRNA-sequencing reads

RNA-sequencing reads used for rumen wall microbial community classification were obtained using the following steps. Total RNA from rumen papilla samples were extracted using miRNeasy kit (Qiagen, US) following manufacturer’s instruction. Extracted total RNAs were further treated with DNase I (Qiagen, US). RNA qualities were assessed using Bioanalyzer RNA 6000 nano kit (Agilent Technologies, US). RNA samples with RIN value > = 8 were pursued for RNA quantification using Qbit (Thermo Fisher, US). RNA-sequencing library preparation was done using Illumina TruSseq ribozero gold kit following manufacturer’s instruction using 1*ug* of total RNA. Using STAR as the alignment tool^72^, RNA-seq reads mapped to the genome of *Bos taurus* (UMD 3.1) were filtered out. To enrich reads coming from microbial rRNA, the remaining, non-cattle RNA-seq raw reads were mapped to rRNA reference databases provided by SortMeRNA^73^ using STAR^72^. Mapped reads were used for downstream microbial taxonomic classification using Kraken^74^, following the protocol here (http://ccb.jhu.edu/software/kraken/MANUAL.html).

To compare the microbial community differences between control and treated groups, taxonomic classifications at the genus level were considered. For each sample, the total number of reads mapped to the each genus level is normalized by the total number of classified reads by Kraken. To do this, the total number of reads mapped to genus level was first divided to 1,000,000, which yields the “per million” factor. Then, the mapped reads at each genus was divided by the “per million factor”, yielding a normalized read count. The statistical significance of differences in genus level abundance between control and treated groups was carried out using non-parametric test, Kruskal-Wallis, by SciPY with *p-value* cutoff of 0.05. For each treatment group, the abundance of each taxon is ranked using averaged, normalized read counts at the genus level. The top 10% most common taxa were compared between treated and control groups. Principal component analysis (PCA) was performed using normalized read count at genus level prcomp in R (version 3.2).

### RT-qPCR verification of target genes

Four genes were analyzed using real time quantitative PCR to assess their differential expression between treated and control groups. These genes are: SGPL1, TMEM175, SMPDL3B and CHST13. SMPDL3B encodes a lipid modifying enzyme, with a reported role in regulating innate immune signaling^53^ and in anti-inflammatory processes^75^. SGPL1 encodes a sphingolipid signaling molecule. TMEM17 encodes a transmembrane protein, with a reported role in regulating luminal pH stability^76^. CHST13 encodes a protein, belonging to sulfotransferase 2 family, and has highly exclusive expression in both fetal and adult liver^77^.

cDNA synthesis was performed using 2000 μg of RNA with High Capacity cDNA master mix (Life technologies). Gene-specific, Taqman assay probes were ordered from Thermo Fisher (Thermo Fisher, USA). All qPCR reactions were performed using the ABI7500 fast system (Applied Biosystems). The thermocycler steps were as follows: one step of uracil-N-glycosylase (UNG)^78,79^ treatment at 50 °C for 2 min, followed by an initial denaturation/activation step at 95 °C for 10 min, then 40 cycles at 95 °C for 15 s and 60 °C for 60 s. The experiments were carried out in triplicate for each data point. The fold change in gene expression was obtained following normalization to two reference genes, Beta-actin (*ACTB*) and hydroxymethylbilane synthase **(***HMBS*). These two reference genes were found to be very consistent in the rumen epithelium^80^. The relative quantification of gene expression was determined using the 2^−ΔΔCt^ method^81^.

## Supplementary information

Supplemental information

Dataset 1

## Data Availability

Gene raw read-counts of liver samples were included in the supplemental dataset. rRNA raw reads obtained from rumen papilla tissues were submitted to NCBI with project accession number of PRJNA478608.
