# Author Correction: Transcriptomics analysis of host liver and meta-transcriptome analysis of rumen epimural microbial community in young calves treated with artificial dosing of rumen content from adult donor cow

**DOI:** 10.1038/s41598-019-44428-4

**Published:** 2019-05-28

**Authors:** Wenli Li, Andrea Edwards, Christina Riehle, Madison S. Cox, Sarah Raabis, Joseph H. Skarlupka, Andrew J. Steinberger, Jason Walling, Derek Bickhart, Garret Suen

**Affiliations:** 1 The Cell Wall Utilization and Biology Laboratory, US Dairy Forage Research Center, USDA ARS, Madison, WI 53706 USA; 2 Department of Biology, University of Wisconsin-Madison, Madison, WI 53706 USA; 3 Department of Genetics, University of Wisconsin-Madison, Madison, WI 53706 USA; 4 Department of Bacteriology, University of Wisconsin-Madison, Madison, WI 53706 USA; 5 Department of Medical Sciences, School of Veterinary Medicine, University of Wisconsin-Madison, Madison, WI 53706 USA; 6 Cereal Crops Research Unit – USDA, 502 Walnut Street Madison, Madison, WI 53726 USA

Correction to: *Scientific Reports* 10.1038/s41598-018-37033-4, published online 28 January 2019

The original version of this Article contained errors in the spelling of the authors Madison S. Cox, Andrew J. Steinberger & Joseph H. Skarlupka which were incorrectly given as Madison Cox, Andrew Steinberger & Joseph Skarlupka respectively.

Additionally, the original version of this Article contained an error in Affiliation 4, which was incorrectly given as ‘Department of Microbiology, University of Wisconsin-Madison, Madison, WI, 53706, USA’. The correct affiliation is listed below:

Department of Bacteriology, University of Wisconsin-Madison, Madison, WI, 53706, USA

Finally, the Acknowledgements section in this Article was incomplete and contained typographical errors.

“We thank Laura Cersoscimo and Rafael Oliveira for their generous help on calf tissue collection. WL and DB were supported by appropriated project 5090-31000-026-00-D from the USDA Agriculture Research Service (Dairy Forage Research Center). JW was supported by appropriated project 5090-43440-006-00-D from the USDA Agricultural Research Service (Cereal Crops Research Laboratory). Mention of trade names or commercial products in this article is solely for the purpose of providing specific information and does not imply recommendation by the US Department of Agriculture. The USDA is an equal opportunity provider and employer.”

now reads:

“We thank Laura Cersosimo and Rafael Oliveira for their generous help on calf tissue collection. WL and DB were supported by appropriated project 5090-31000-026-00-D from the USDA Agriculture Research Service (Dairy Forage Research Center). JW was supported by appropriated project 5090-43440-006-00-D from the USDA Agricultural Research Service (Cereal Crops Research Laboratory). GS was supported by, in part, by a USDA National Institute of Food and Agriculture HATCH grant WIS02007 and a USDA Agriculture and Food Research Initiative Competitive Grant no. 2015-67015-23246. MSC was supported by a USDA Agriculture and Food Research Initiative Education and Literacy Initiative predoctoral fellowship no. 2018-67011-27997. Mention of trade names or commercial products in this article is solely for the purpose of providing specific information and does not imply recommendation by the US Department of Agriculture. The USDA is an equal opportunity provider and employer.”

These errors have now been corrected in the PDF and HTML versions of the Article, and in the accompanying Supplementary Information file.

