# Supplementary material for: Transcriptomics analysis of host liver and meta-transcriptome analysis of rumen epimural microbial community in young calves treated with artificial dosing of rumen content from adult donor cow
Source: Sci Rep. 2019 Jan 28;9:790. doi: 10.1038/s41598-018-37033-4 (PMC6349911; doi:10.1038/s41598-018-37033-4)
Supplement: Supplementary file 1 — Supplemental information [file 41598_2018_37033_MOESM1_ESM.docx]

Supplementary Information

Title: **Transcriptomics analysis of host liver and meta-transcriptome analysis of rumen epimural microbial community in young calves treated with artificial dosing of rumen content from adult donor cow**

Wenli Li^1^, Andrea Edwards^2^, Christina Riehle^3^, Madison S. Cox^4^, Sarah Raabis^5^, Joseph H. Skarlupka^4^, Andrew J. Steinberger^4^, Jason Walling^6^ Derek Bickhart^1^ and Garret Suen^4^

^1^The Cell Wall Utilization and Biology Laboratory, US Dairy Forage Research Center, USDA ARS, Madison, WI, 53706, USA

^2^Department of Biology, University of Wisconsin-Madison, Madison, WI, 53706, USA

^3^Department of Genetics, University of Wisconsin-Madison, Madison, WI, 53706, USA

^4^Department of Bacteriology, University of Wisconsin-Madison, Madison, WI, 53706, USA

^5^Department of Medical Sciences, School of Veterinary Medicine, University of Wisconsin-Madison, Madison, WI, 53706, USA

^6^Cereal Crops Research Unit – USDA, 502 Walnut Street Madison, WI, 53726, USA

Corresponding author: Wenli Li, US Dairy Forage Research Center. email: wenli.li@ars.usda.gov

**Supplemental Table 1.** Total number of expressed genes in each FPKM category.

| **Liver samples** | **fpkm>=500** | **100<=fpkm<500** | **15<=fpkm<100** | **0.2<fpkm<15** | **total # of expressed genes** | **total # genes assessed** | **Treatment** |
| --- | --- | --- | --- | --- | --- | --- | --- |
| 6773 | 49 | 223 | 1021 | 12375 | 13668 | 28538 | Treated |
| 6780 | 74 | 291 | 1379 | 12543 | 14287 | 28538 | Control |
| 6775 | 58 | 257 | 1217 | 12545 | 14077 | 28538 | Treated |
| 6792 | 62 | 280 | 1278 | 12386 | 14006 | 28538 | Control |
| 6793 | 58 | 261 | 1151 | 12389 | 13859 | 28538 | Treated |
| 6771 | 70 | 310 | 1380 | 12125 | 13885 | 28538 | Control |
| 6768 | 55 | 248 | 1096 | 12270 | 13669 | 28538 | Treated |
| 6766 | 84 | 335 | 1612 | 12237 | 14268 | 28538 | Control |

**Supplemental Table 2.** The list of significantly differentially expressed genes.

| **Gene** | **baseMean** | **log2FoldChange (Treated vs control)** | **adjusted pvalue** |
| --- | --- | --- | --- |
| LOC101906884 | 586.936424 | 1.54327762 | 0.000125694 |
| CHST13 | 31.9011816 | 1.46146471 | 5.28E-07 |
| LOC515674 | 14.2053958 | 1.402155831 | 0.000148869 |
| STON2 | 2.60792773 | 1.242408666 | 0.0021211 |
| ZBTB46 | 2.96229176 | 1.187870576 | 0.00332207 |
| LOC101904426 | 3.96226574 | 1.166317336 | 0.005168386 |
| LOC101908002 | 47.5933087 | 1.149767419 | 5.62E-05 |
| FAM20A | 6.74561486 | 1.147405441 | 0.005054077 |
| LOC100138449 | 6.2796629 | 1.142326803 | 0.004956832 |
| SMPDL3B | 60.3375498 | 1.130454446 | 0.000120196 |
| ANKRD11 | 24.9807558 | 1.119613067 | 0.000219196 |
| SGSM2 | 3.99054139 | 1.10308 | 0.008232875 |
| TPD52 | 6.31420048 | 1.099956685 | 0.006536182 |
| NIPSNAP3A | 2.70846587 | 1.079981389 | 0.00851493 |
| TMEM41A | 16.4261678 | 1.078170981 | 0.003537551 |
| DOCK6 | 18.3582415 | 1.070631672 | 0.003701248 |
| POLR3C | 9.68326069 | 1.05526311 | 0.005513341 |
| LOC786942 | 7.64879475 | 1.05089795 | 0.008939791 |
| LOC101906246 | 497693.762 | 1.041501835 | 0.012062074 |
| LOC101902223 | 7.73009372 | 1.039168986 | 0.009349549 |
| LOC101905652 | 61.8277976 | 1.038888021 | 0.000827067 |
| KNOP1 | 5.7172812 | 1.014238908 | 0.014758909 |
| RNASEK | 3.74014616 | 1.009386226 | 0.015536807 |
| SGPL1 | 99.646743 | 1.007272868 | 8.12E-05 |
| ESYT1 | 23.1663855 | 1.000105972 | 0.004161282 |
| HTR2A | 3.34511959 | 0.999326843 | 0.014912432 |
| FLRT3 | 25.619117 | 0.999251306 | 0.001961304 |
| LOC101904132 | 8.08505778 | 0.992247168 | 0.01525791 |
| LOC101907101 | 6.34131789 | 0.98811143 | 0.014535018 |
| LOC101907057 | 14.2625629 | 0.987286434 | 0.006714829 |
| LOC100295848 | 6.93654236 | 0.984675777 | 0.015614218 |
| LOC101906346 | 356480.568 | 0.98373769 | 0.018450075 |
| ZNF295 | 4.36192161 | 0.97908421 | 0.018569741 |
| LOC101906401 | 667266.72 | 0.968486183 | 0.020350131 |
| CYGB | 8.60691046 | 0.968416858 | 0.014987984 |
| FAT2 | 8.27636784 | 0.961109669 | 0.016400104 |
| LOC101906075 | 8.01521419 | 0.959454407 | 0.021534997 |
| LOC101904117 | 27783.9743 | 0.954905169 | 0.021057412 |
| HSPA1A | 30.2116703 | 0.952678609 | 0.008141875 |
| LOC101905846 | 3510391.59 | 0.951041844 | 0.020045835 |
| C17H22orf13 | 78.565008 | 0.950226955 | 0.007544094 |
| TDO2 | 9.16347064 | 0.948999168 | 0.013419064 |
| SCGN | 60.1592354 | 0.946550075 | 0.000666243 |
| GIN1 | 22.1312522 | 0.946199528 | 0.006444315 |
| LOC101904705 | 5.2155657 | 0.94200918 | 0.023548246 |
| NAMPT | 79.0117177 | 0.932919471 | 0.001814876 |
| FHIT | 8.12902027 | 0.931240818 | 0.009716683 |
| CUX2 | 5.43617396 | 0.927228901 | 0.024026856 |
| IL17B | 11.4411588 | 0.927075421 | 0.011544838 |
| ASXL1 | 2.07671863 | 0.923973004 | 0.020099365 |
| CYBASC3 | 23.9577245 | 0.921984114 | 0.002055287 |
| TMEM175 | 127.471596 | 0.921927062 | 0.006221402 |
| VPS37A | 19.6480724 | 0.917845365 | 0.017607211 |
| APMAP | 4.71223839 | 0.914374994 | 0.027370961 |
| LOC101903893 | 2.63082979 | 0.91317341 | 0.026049242 |
| LYPLA2 | 10.5290064 | 0.912178693 | 0.016861569 |
| MYO7B | 5.24092751 | 0.909600108 | 0.029083243 |
| LOC100851064 | 4.69099861 | 0.909006487 | 0.02939435 |
| TARBP2 | 1.48248421 | 0.907441964 | 0.012547197 |
| HSPA12A | 5.25526373 | 0.906295151 | 0.02827549 |
| MPP1 | 2.72237695 | 0.899614973 | 0.027346547 |
| LOC101903393 | 8.61315426 | 0.89665849 | 0.021540075 |
| LOC100847981 | 5.50666926 | 0.896509814 | 0.030240711 |
| HCN2 | 16.9965998 | 0.89395812 | 0.007703146 |
| CHDH | 8.13237326 | 0.892107651 | 0.027391658 |
| LOC101905792 | 120.779725 | 0.891530792 | 0.003359456 |
| INO80E | 9.29486689 | 0.890155214 | 0.02098797 |
| LOC101903292 | 51.5827995 | 0.88290365 | 0.004808755 |
| CTU2 | 2.16121546 | 0.882520385 | 0.02854502 |
| LOC101908091 | 21.9261719 | 0.882253322 | 0.011565056 |
| DLEC1 | 327.857796 | 0.877255952 | 0.000214538 |
| LOC101903739 | 2.00130815 | 0.872520149 | 0.014707499 |
| TNK1 | 7.8392909 | 0.871771512 | 0.027829197 |
| CASKIN2 | 20.090253 | 0.871535381 | 0.00718884 |
| LOC101901899 | 6.50119922 | 0.869150731 | 0.031787236 |
| RFC1 | 60.9674918 | 0.867356507 | 0.015796649 |
| PACSIN3 | 2.52403641 | 0.865445829 | 0.030713684 |
| DSG2 | 1.94952119 | 0.864857052 | 0.021481992 |
| ZNF391 | 3.41726532 | 0.86335303 | 0.038465595 |
| SHISA5 | 6.33242344 | 0.859291161 | 0.038045001 |
| RHBDL1 | 3.80849083 | 0.852280495 | 0.04079788 |
| LOC101904940 | 42.1878235 | 0.85208718 | 0.024110248 |
| FMO5 | 12.161501 | 0.852058464 | 0.031673744 |
| OXER1 | 2.23469038 | 0.850512946 | 0.035013256 |
| C13H20orf94 | 1.6685538 | 0.844785356 | 0.023897893 |
| ZBTB17 | 3.71960013 | 0.844629348 | 0.041239914 |
| LOC101904038 | 38241.217 | 0.843706081 | 0.022865776 |
| LOC523963 | 6.66154004 | 0.838921764 | 0.041404262 |
| PARP2 | 1.90593407 | 0.837763728 | 0.033342648 |
| LOC530739 | 44.2479638 | 0.833977662 | 0.002689 |
| LOC786073 | 4.96613702 | 0.832915076 | 0.044346416 |
| LOC101904711 | 6.17329267 | 0.832210487 | 0.04022713 |
| NDUFA3 | 3.13766754 | 0.831165948 | 0.043859546 |
| RASSF4 | 112.596584 | 0.830677678 | 0.042112688 |
| LOC101904964 | 18.4534271 | 0.830328453 | 0.01404842 |
| LOC101902410 | 37.6055678 | 0.828506286 | 0.00336224 |
| LOC101902408 | 1.4570809 | 0.826777211 | 0.02739899 |
| ECM2 | 8.55519296 | 0.82318394 | 0.033413367 |
| UROC1 | 2.33095585 | 0.819197046 | 0.045867279 |
| ATP2A1 | 6.08401924 | 0.819087962 | 0.042884704 |
| ZC3H7B | 6.91812376 | 0.81906293 | 0.044425383 |
| JUNB | 2.99970698 | 0.819001562 | 0.046411735 |
| USMG5 | 8.23396502 | 0.815587781 | 0.034356715 |
| ATP6V1E1 | 5.40629592 | 0.815007594 | 0.049177638 |
| LOC101904104 | 30.0087235 | 0.813541411 | 0.005026646 |
| PLIN4 | 1.63608286 | 0.810169581 | 0.028085393 |
| BCL2L13 | 4.54067148 | 0.809804057 | 0.05139268 |
| RHBDF1 | 6.04585837 | 0.806965034 | 0.047311584 |
| PNPLA6 | 2.65054838 | 0.806776835 | 0.051211105 |
| FIGNL2 | 4.41432434 | 0.806487103 | 0.051450689 |
| RPL12 | 39.1154487 | 0.805356203 | 0.003316816 |
| SNTB1 | 7.95299187 | 0.804898602 | 0.046475792 |
| HACL1 | 13.2730473 | 0.804706205 | 0.03442119 |
| LOC101906524 | 279.445784 | 0.79767881 | 0.000667922 |
| MSTO1 | 10.0094998 | 0.796166396 | 0.032535897 |
| ILDR1 | 7.66133593 | 0.79342752 | 0.051329047 |
| ZNF789 | 3.33481159 | 0.787417911 | 0.055498151 |
| HNRNPH3 | 15.3573524 | 0.786418677 | 0.03195984 |
| APEG3 | 10.7154359 | 0.786116279 | 0.029877798 |
| PRRT3 | 1.74834781 | 0.785564947 | 0.043292639 |
| LOC101907319 | 8.88954432 | 0.784968137 | 0.044603978 |
| FILIP1L | 31.1734898 | 0.784529376 | 0.003809217 |
| LOC101905639 | 31.3915424 | 0.783824309 | 0.023497462 |
| TIMP3 | 40.5522511 | 0.783541922 | 0.014187151 |
| C1H3orf38 | 1.78521338 | 0.776360018 | 0.043325041 |
| LRRC16A | 170.864897 | 0.772631215 | 0.011000776 |
| FAM151A | 8.48636845 | 0.771178382 | 0.047846555 |
| CYP17A1 | 22.3904611 | 0.770897457 | 0.01463004 |
| TMEM140 | 23.8396374 | 0.762552495 | 0.011675259 |
| LOC101904892 | 135.868514 | 0.758608249 | 0.008780562 |
| CLDN7 | 25.3188293 | 0.756566753 | 0.026102948 |
| PLS1 | 15.7916391 | 0.754291917 | 0.031950856 |
| LOC101905441 | 29.838329 | 0.751949643 | 0.020561719 |
| CD74 | 17.7454636 | 0.751150774 | 0.018416887 |
| HOMEZ | 9.6008096 | 0.740677578 | 0.050854233 |
| LOC101906586 | 575.21509 | 0.740324392 | 0.008767269 |
| LOC101905916 | 13.3746981 | 0.737105241 | 0.044238282 |
| LOC101904528 | 21.9941699 | 0.73646576 | 0.032952744 |
| FICD | 20.5423456 | 0.735488161 | 0.024949866 |
| LOC101903555 | 12.1434768 | 0.733738612 | 0.04199629 |
| LOC101902399 | 24.6063495 | 0.732820312 | 0.021775497 |
| LOC101902157 | 14.5417239 | 0.732382132 | 0.02732612 |
| THRA | 20.7357277 | 0.731375891 | 0.034236859 |
| LOC101906796 | 20.1624083 | 0.729786973 | 0.023685435 |
| FGFR3 | 1.16060544 | 0.729175971 | 0.032245698 |
| TAGLN | 1.06864187 | 0.726682104 | 0.03277098 |
| LOC101905083 | 23.5822798 | 0.724741812 | 0.02252028 |
| KIAA1984 | 12.9434322 | 0.723198324 | 0.05266997 |
| TC2N | 1.39178551 | 0.721872693 | 0.048817415 |
| IFT46 | 105.079887 | 0.715902294 | 0.013976086 |
| LOC101906437 | 176.308702 | 0.697084231 | 0.006686853 |
| SRSF12 | 19.2544154 | 0.692211596 | 0.032563909 |
| HNF1A | 62.6478955 | 0.690939926 | 0.002188692 |
| LOC101902723 | 122.334996 | 0.690871352 | 0.002154454 |
| LOC100852241 | 20.9176327 | 0.687858918 | 0.033135176 |
| SLC24A2 | 20.8994788 | 0.686709224 | 0.028379892 |
| SLC26A1 | 58.2774707 | 0.686590231 | 0.017058423 |
| LOC539106 | 24.341515 | 0.683258169 | 0.026434692 |
| SLC29A1 | 19.3396209 | 0.679056809 | 0.043330131 |
| CDC42SE2 | 42.3644171 | 0.674499387 | 0.01820169 |
| LOC100848533 | 84.8602637 | 0.670724704 | 0.007964323 |
| ANKS3 | 54.7732157 | 0.66996091 | 0.027193381 |
| PIEZO1 | 16.9351976 | 0.666679763 | 0.045152019 |
| GATAD2A | 0.9557734 | 0.663122896 | 0.044725218 |
| LOC100848639 | 18.0497499 | 0.656738662 | 0.044575961 |
| DFNA5 | 14.7016511 | 0.654732788 | 0.053608111 |
| FAM116B | 31.3444492 | 0.65139723 | 0.033549674 |
| LOC101905950 | 89.9553136 | 0.646816973 | 0.016529695 |
| HIST1H2BA-2 | 27.9250279 | 0.644874813 | 0.043521643 |
| LOC619094 | 0.9462345 | 0.644668794 | 0.048747458 |
| SPSB2 | 19.8497426 | 0.641654917 | 0.047143002 |
| KALRN | 27.7016024 | 0.637066952 | 0.020604506 |
| LOC101905655 | 102.078143 | 0.636101027 | 0.013816303 |
| LOC101902707 | 164.708842 | 0.629814188 | 0.007699499 |
| TOR1AIP2 | 32.8338762 | 0.629235641 | 0.026001302 |
| PVR | 90.8634943 | 0.624624546 | 0.022725263 |
| LOC101906083 | 52.8914288 | 0.623873452 | 0.031827497 |
| ENG | 22.2032075 | 0.621763614 | 0.04468763 |
| TRO | 41.1888462 | 0.619550024 | 0.018407585 |
| LOC101905358 | 40.1596965 | 0.617508307 | 0.017667483 |
| PCK2 | 19.6982872 | 0.61524318 | 0.049436842 |
| LOC101904591 | 21.9379351 | 0.615162742 | 0.044386127 |
| LOC100296121 | 24.1397988 | 0.612298832 | 0.033646317 |
| FAM96B | 296.594882 | 0.611178703 | 0.014223651 |
| LOC100848335 | 51.1190408 | 0.610783717 | 0.021350136 |
| SLC17A4 | 311.591535 | 0.605877504 | 0.025192259 |
| PIH1D1 | 78.0079942 | 0.605162991 | 0.007200271 |
| PRSS48 | 76.0197918 | 0.604253928 | 0.008077962 |
| C10H15orf62 | 91.1183205 | 0.60057582 | 0.005404773 |
| MPP7 | 678.372278 | 0.595028467 | 0.002392462 |
| STX4 | 50.07462 | 0.594722813 | 0.013571832 |
| ZMYND15 | 86.7842877 | 0.587424205 | 0.026137481 |
| LOC101906335 | 46.3061837 | 0.583152336 | 0.017396608 |
| MYH10 | 60.8387042 | 0.581522073 | 0.017471889 |
| IRF1 | 36.422648 | -0.57971905 | 0.049877168 |
| MTF2 | 34.3838775 | -0.581982272 | 0.054437045 |
| EXOSC3 | 40.6548211 | -0.58258736 | 0.024114505 |
| CALR | 62.2197752 | -0.587172465 | 0.054006179 |
| SPG11 | 47.9760458 | -0.590531077 | 0.021616983 |
| FGB | 444.700446 | -0.592629509 | 0.044261373 |
| LOC101905455 | 138.601128 | -0.600209514 | 0.001668491 |
| CORIN | 36.0577089 | -0.606983458 | 0.031869759 |
| FGD6 | 125.747552 | -0.611558614 | 0.004714538 |
| CEBPZ | 145.254269 | -0.613597689 | 0.00351168 |
| LOC101902711 | 75.7123597 | -0.614379698 | 0.00957127 |
| RPAIN | 138.540543 | -0.615777849 | 0.000527609 |
| GLS | 169.55456 | -0.61590572 | 0.006642813 |
| FCHSD2 | 29.9792556 | -0.61782169 | 0.047516308 |
| SF1 | 43.8706625 | -0.623981122 | 0.010987402 |
| PPT1 | 31.0938796 | -0.62508853 | 0.035912327 |
| FGG | 230.822831 | -0.628919576 | 0.03 |
| CAST | 49.7879386 | -0.629598859 | 0.030453632 |
| ILF2 | 44.7236713 | -0.630148801 | 0.011187994 |
| PDE6A | 20.5836351 | -0.641076187 | 0.042331269 |
| LOC101906291 | 215058.128 | -0.646079958 | 0.045525973 |
| CEP290 | 290.951089 | -0.647224711 | 0.000412094 |
| DONSON | 19.1673634 | -0.647890899 | 0.055498623 |
| MIF4GD | 45.2563172 | -0.650162032 | 0.008600648 |
| GPR44 | 68.4142451 | -0.654147412 | 0.018429644 |
| LOC101904069 | 34.330737 | -0.659528735 | 0.023970079 |
| RN18S1 | 86421.917 | -0.661033366 | 0.042495627 |
| LOC101902332 | 88.5559966 | -0.661698489 | 0.005958564 |
| ATXN2 | 20.4824148 | -0.662762007 | 0.050900841 |
| SFRS13A | 40.2116087 | -0.666127642 | 0.014484429 |
| LOC101907298 | 14.5940017 | -0.669867693 | 0.050952681 |
| TIMM22 | 48.9183632 | -0.687089743 | 0.006399212 |
| NRM | 90.5229252 | -0.687240327 | 0.008605168 |
| ATP1B1 | 38.0522076 | -0.687411731 | 0.021501313 |
| APOA5 | 21.1250233 | -0.694437357 | 0.045708157 |
| C5H12orf39 | 17.9943658 | -0.706351302 | 0.048590774 |
| ANGEL2 | 1.14028593 | -0.70896967 | 0.040358442 |
| B3GNT9 | 77.1276459 | -0.709199527 | 0.013206457 |
| SLC35A3 | 20.7959078 | -0.711495164 | 0.041226206 |
| C6H4orf3 | 34.2363002 | -0.712845446 | 0.034539259 |
| HDAC1 | 13.1540812 | -0.713534064 | 0.051487203 |
| MTFR1 | 32.6726651 | -0.714525884 | 0.018517897 |
| LOC101905751 | 13.7552998 | -0.715318014 | 0.053890916 |
| FSD1L | 1.1766269 | -0.715427949 | 0.038858214 |
| NID2 | 15.117419 | -0.716858315 | 0.040224668 |
| RPL4 | 22.8536637 | -0.717683385 | 0.021668983 |
| ZNF672 | 9.73986278 | -0.731824735 | 0.054792737 |
| SPON2 | 1.70126104 | -0.733918456 | 0.047715688 |
| RRP36 | 1.5959731 | -0.734245112 | 0.049259966 |
| LOC100196901 | 10.1089973 | -0.735861232 | 0.044574084 |
| LOC100300956 | 71.6550429 | -0.739434994 | 0.000604672 |
| LOC790312 | 21.8130921 | -0.740579381 | 0.052218759 |
| LOC516599 | 22.1679456 | -0.741793305 | 0.016001643 |
| ARHGEF6 | 2.18889658 | -0.747550089 | 0.051665773 |
| TAPBP | 25.9494047 | -0.750744947 | 0.031314342 |
| LOC101904193 | 55273.6158 | -0.754886577 | 0.026448505 |
| FYN | 1.97688949 | -0.754912317 | 0.05284007 |
| PNMAL1 | 70.2870399 | -0.761709518 | 0.006513613 |
| JAK2 | 15.2236333 | -0.764789122 | 0.040229054 |
| MRPS36 | 7.69900066 | -0.770433949 | 0.053889509 |
| ZSWIM1 | 15.2898075 | -0.771255529 | 0.027876157 |
| MMACHC | 10.1341326 | -0.773216166 | 0.039755309 |
| LOC521656 | 31.6200526 | -0.7761459 | 0.036139548 |
| HNRNPL | 2.27207204 | -0.776183663 | 0.044528632 |
| CASP6 | 20.9649133 | -0.779055301 | 0.019235793 |
| REST | 2.1213546 | -0.784559004 | 0.045115626 |
| PYGL | 18.8665482 | -0.787671885 | 0.019364201 |
| LOC101904159 | 18952.7575 | -0.793703202 | 0.021510772 |
| LOC101903658 | 7.09891577 | -0.793775188 | 0.05402446 |
| ICK | 9.78048843 | -0.793975937 | 0.053637168 |
| LOC514978 | 4.6206595 | -0.795619019 | 0.054650763 |
| TMEM165 | 6.4835893 | -0.796858817 | 0.051690576 |
| LOC100847664 | 31.7239897 | -0.797385648 | 0.005036165 |
| NANOS1 | 13.684754 | -0.80431106 | 0.023689053 |
| DTX3 | 2.01904472 | -0.807108526 | 0.043181555 |
| PRKRIR | 3.18819264 | -0.807306913 | 0.045011562 |
| BRF2 | 2.86376202 | -0.808033765 | 0.045347584 |
| LOC101903912 | 8.69901139 | -0.811089126 | 0.041481581 |
| ATP6V1D | 13.6017051 | -0.815533534 | 0.023477623 |
| MRP63 | 13.013069 | -0.817991825 | 0.018585702 |
| HSD17B6 | 7.26091057 | -0.821853422 | 0.047211689 |
| SLC39A7 | 3.64388488 | -0.833942736 | 0.045721429 |
| SIAE | 9.19964598 | -0.834858704 | 0.027043502 |
| PSTPIP2 | 3.28634447 | -0.835402841 | 0.044513675 |
| RANBP6 | 1.82220477 | -0.838464045 | 0.02939429 |
| FBXO21 | 10.7487622 | -0.840890166 | 0.028238861 |
| TET1 | 11.3668923 | -0.842494673 | 0.030731905 |
| IWS1 | 2.17963927 | -0.846248291 | 0.034455612 |
| LOC788414 | 16.3798805 | -0.848131717 | 0.018932052 |
| BRD7 | 10.4578715 | -0.853520032 | 0.024057225 |
| U2AF1 | 3.3547368 | -0.854918056 | 0.039396165 |
| ZBTB25 | 30.892117 | -0.857451859 | 0.001573782 |
| FGFR1 | 3.11936575 | -0.85758793 | 0.03822274 |
| ANKRD35 | 3.52181924 | -0.858771883 | 0.03895309 |
| HSD17B13 | 7.37069587 | -0.865658476 | 0.031254761 |
| RPS3 | 3.46903744 | -0.869818644 | 0.036725798 |
| RNF146B | 26.8065827 | -0.870559851 | 0.005068464 |
| GABPB1 | 12.2916592 | -0.882775111 | 0.024378374 |
| LGALS1 | 2.24247198 | -0.882823448 | 0.028194673 |
| TM4SF4 | 2.7383713 | -0.886831999 | 0.030303691 |
| SEC16A | 19.723359 | -0.887197834 | 0.020007905 |
| COMMD10 | 3.90354085 | -0.889061494 | 0.033203303 |
| LOC101905061 | 5.62120633 | -0.892678122 | 0.030758501 |
| PTAR1 | 1.91729182 | -0.898713466 | 0.021694955 |
| USP53 | 39.690416 | -0.904143448 | 0.000563133 |
| PHB2 | 2.25824986 | -0.905592653 | 0.024669098 |
| PEBP1 | 10.0300049 | -0.908092703 | 0.026261362 |
| DUSP6 | 12.3050824 | -0.912577481 | 0.014973614 |
| EP400 | 6.29423476 | -0.919666004 | 0.02462628 |
| CTAGE5 | 13.9359579 | -0.923780671 | 0.012186672 |
| HCLS1 | 3.52780926 | -0.92700814 | 0.026208794 |
| MAP3K6 | 5.46500859 | -0.943481479 | 0.022673776 |
| HSPBAP1 | 9.12682388 | -0.948917065 | 0.015213025 |
| EFCAB14 | 12.4034492 | -0.950904002 | 0.019266415 |
| SULT2A1 | 14.3964817 | -0.951660981 | 0.008038755 |
| LOC101907512 | 18.4329177 | -0.957876006 | 0.003672096 |
| INSR | 2.03655939 | -0.958965189 | 0.014606249 |
| CMTM6 | 7.51906227 | -0.976803877 | 0.01551 |
| HMMR | 16.6652759 | -0.977288635 | 0.008321484 |
| LOC100847417 | 46.4321763 | -0.98148052 | 0.000884333 |
| FAM70A | 8.72775105 | -0.987248166 | 0.01099958 |
| ZNF140 | 5.84672168 | -0.988202809 | 0.015530932 |
| ECD | 9.4901439 | -0.993634481 | 0.013672232 |
| LOC100140261 | 7.66256458 | -1.006315605 | 0.012663702 |
| SAP18 | 7.47739911 | -1.012108245 | 0.01230506 |
| HBS1L | 15.1267215 | -1.012833469 | 0.003572907 |
| ATP10B | 5.19627562 | -1.013463443 | 0.014699692 |
| TEX2-2 | 31.3034148 | -1.034797069 | 0.000243755 |
| AP1AR | 21.0068484 | -1.041484633 | 0.001122924 |
| HMGXB3 | 15.2235809 | -1.046024391 | 0.003761652 |
| CHCHD10 | 1.9140196 | -1.074563815 | 0.005406567 |
| TRNAU1AP | 3.86590424 | -1.082869857 | 0.009442713 |
| LOC101906747 | 12.7317172 | -1.103877082 | 0.001723152 |
| BMP2K | 52.045175 | -1.10489309 | 0.000668692 |
| NQO2 | 2.36901095 | -1.107932657 | 0.005923683 |
| LOC101904088 | 2806.87542 | -1.108274272 | 0.004712199 |
| EHHADH | 10.578576 | -1.140161627 | 0.006314088 |
| POGZ | 3.71916733 | -1.15571898 | 0.005265937 |
| LIPM | 13.4497056 | -1.167355787 | 0.002275513 |
| GNRH2 | 3.97669184 | -1.192294586 | 0.004286418 |
| UGT2B4 | 3.32070891 | -1.250702566 | 0.002466655 |
| LOC514658 | 42.0632206 | -1.252211646 | 0.001029254 |
| LOC790886-2 | 10.7658592 | -1.33040634 | 0.000760626 |
| MAPK6 | 6.51210362 | -1.470149893 | 0.00037954 |

**Supplemental Table 3. Expression of genes involved in innate immune response.**

| **Gene name** | **baseMean** | **log2FoldChange** | **pvalue** | **Tissue expression** |
| --- | --- | --- | --- | --- |
| CD74 | 17.74 | 0.75 | 0.02 | Liver spleen |
| JAK2 | 15.22 | -0.76 | 0.04 | non-specific |
| EXOSC3 | 40.65 | -0.58 | 0.02 | non-specific |
| FGB | 444.70 | -0.59 | 0.04 | Liver only |
| FGG | 230.82 | -0.63 | 0.03 | Liver only |
| IRF1 | 36.42 | -0.58 | 0.05 | Non-specific |
| PVR | 90.86 | 0.62 | 0.02 | Non-specific |
| SMPDL3B | 60.33 | 1.13 | 0.0001 | small intestine, thyroid, pancreas, stomach, colon |

**Supplemental Figure 1a**. The expression of *SMPDL3B* in human tissues.


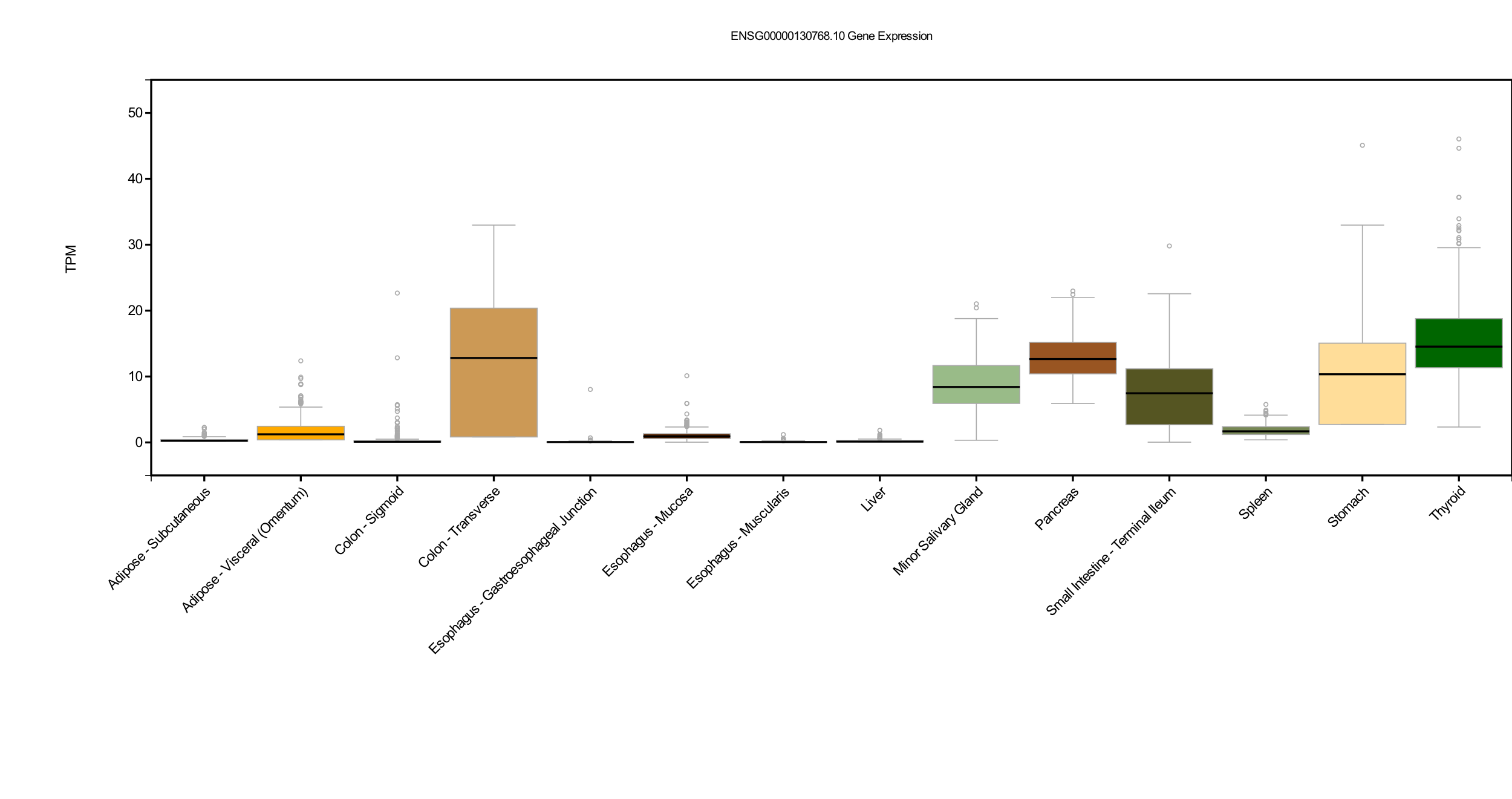


**Supplemental Figure 1b**. The expression of *SMPDL3B* in cattle (*Bos taurus*) tissues. This image is obtained from the Expression Atlas^1^ by European Bioinformatics Institute under a [Creative Commons Attribution 4.0 International License](http://creativecommons.org/licenses/by/4.0/) (https://www.ebi.ac.uk/gxa/licence.html).


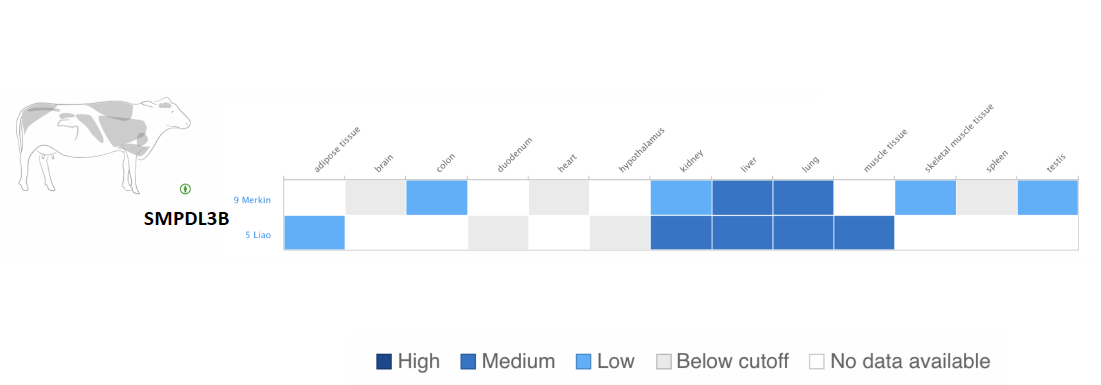


**Supplemental Figure 2a**. The expression of *FGG* and *FGB* in human tissues. Top figure represents the expression of FGB while bottom figure represents the expression of FGG.


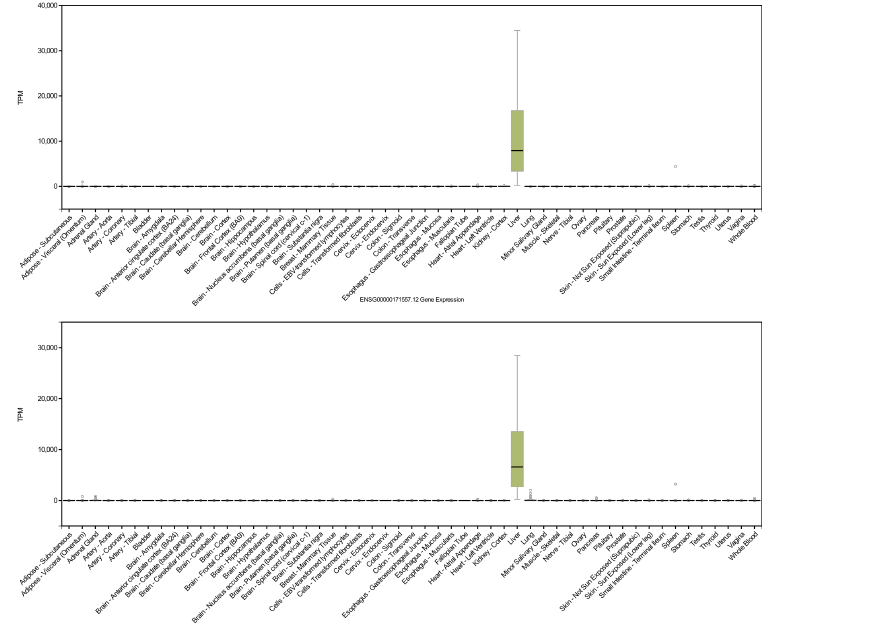


**Supplemental Figure 2b**. The expression of *FGG* and *FGB* in cattle (*Bos taurus*) tissues. Top figure represents the expression of FGB while bottom figure represents the expression of FGG. This image is obtained from the Expression Atlas^1^ by European Bioinformatics Institute under a [Creative Commons Attribution 4.0 International License](http://creativecommons.org/licenses/by/4.0/) (https://www.ebi.ac.uk/gxa/licence.html).


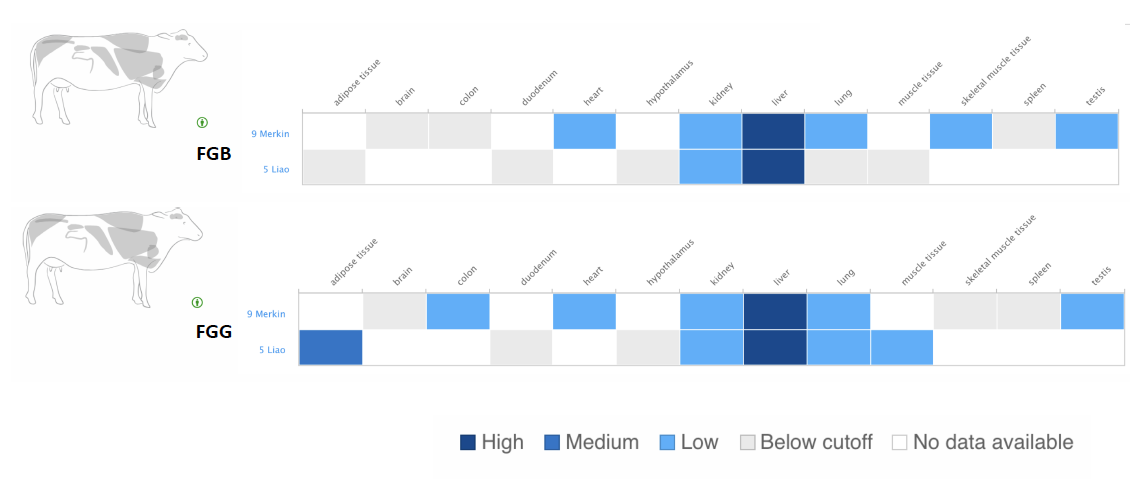


**Reference**

1 Petryszak, R. *et al.* Expression Atlas update--an integrated database of gene and protein expression in humans, animals and plants. *Nucleic Acids Res* **44**, D746-752, doi:10.1093/nar/gkv1045 (2016).
